# Assessment of the readiness and response toward the dengue fever outbreak (2019) in Sudan: a qualitative exploration

**DOI:** 10.1186/s12889-023-17020-9

**Published:** 2023-10-30

**Authors:** Abdulla M. Bagahizel, Hamadnalla Sir Elkhatim

**Affiliations:** 1 College of Medicine, Hadhramout University, Mukalla, Yemen; 2 University of Medical Sciences and Technology, Khartoum, Sudan

**Keywords:** Dengue fever, Outbreak, Readiness assessment, Response assessment, Sudan

## Abstract

**Background:**

Dengue fever (DF) is a mosquito-borne viral disease transmitted by *Aedes* mosquito species and has been considered a major public health problem in Kassala State for tens of years. This study aimed to assess the level of readiness and response toward the 2019 dengue fever outbreak in Kassala at the state and community levels.

**Methods:**

This exploratory cross-sectional study was conducted in Kassala State, Sudan, from January to March 2020. The researcher conducted interviews with the key respondents at the state level to assess the level of readiness and response and to reflect the capacity of institutions—public health authorities, health systems, and emergency response bodies.

**Results:**

The surveillance system reported 3961 DF cases in Kassala State, representing 93.5% of the total cases in Sudan between August 2019 and January 2020. This outbreak was identified by passive surveillance, 51 samples were tested during the outbreak period, and private clinics and labs were not included in the surveillance system. According to the WHO checklist of outbreak readiness and response, Kassala's surveillance system and public health laboratory received the lowest scores.

**Conclusions:**

The study concludes that outbreak readiness and response could be considered below standards, mainly in the surveillance system and laboratory diagnostic facilities, due to the absence of intersectoral collaboration with a regulatory framework in terms of financial and operational participation.

**Supplementary Information:**

The online version contains supplementary material available at 10.1186/s12889-023-17020-9.

## Background

Dengue fever (DF) is a neglected tropical disease that affects a large number of endemic countries and has a risk for severe manifestations, such as dengue hemorrhagic fever. The incidence of dengue has risen in the last half-century by 30-fold [1]. Up to 400 million people become infected every year, while approximately 100 million become sick from infection, an estimated 500,000 people with severe dengue require hospitalization and 22,000 people die from severe dengue [2–4].

Rapid responses to dengue outbreaks are required to control the spread of the infection and to address the high number of cases. A wide range of different interventions has been employed to meet these demands [5]. Preparedness planning (used synonymously with outbreak response planning or contingency planning) has been described as a manner by which to expand the commitment of partners, build capacity and develop infrastructure, and give operational links to ensure an organized and facilitated response [6]. Emergency preparedness and anticipated response planning are an integral part of dengue control, yet this is often neglected in dengue-endemic countries. Various measures need to be conducted according to the context of dengue in the area. Hence, in endemic areas, the ability to identify and coordinate an outbreak response should be a priority, while in dengue-free areas, procedures depend on responding to sporadic cases, risk indicators, or alert signals [7]. Studies that assess outbreak responses have been difficult to interpret as they generally describe a speared scope of interventions applied in various ways, and the available literature mainly concentrates on epidemiological surveillance or vector control. A global research agenda set up by the Scientific Working Group at the WHO in 2006 occurred due to a lack of evidence-based outbreak response strategies, and it was recommended that case studies of national programs to recognize factors prompting the success or failure of dengue control and prevention programs. It has also been recommended that future research should compare national and international policies in emergency response plans to identify the common interventions currently described [8]. Moreover, a systematic review emphasized the significance of community participation, selective spraying of premises, and environmental management through systematic searching and demolition teams for the successful control of a dengue outbreak [9]. Another studies highlighted the involvement of key stakeholders in dengue control across ten documents, with an informal emphasis on the roles of two specific stakeholders [10, 11].

In Sudan, mosquito-borne viruses including dengue have been considered a major public health problem, predominantly in Kassala during the last 10 years. Recently in August 2019, a dengue fever outbreak occurred in Sudan. In November 2019 the WHO reported a total of 1,197 suspected cases of dengue fever in Sudan (1,111 cases were in Kassala only) from 8th August through 4th November 2019, including five deaths from seven states [12]. The report of Sudan Federal Ministry of Health on 31st January 2020 revealed that most of the cumulative dengue fever cases and deaths were in Kassala [13]. Before less than one year, another mosquito-borne virus outbreak occurred in Kassala and Red Sea States (Chikungunya outbreak in 2018—2019) with a total of 48,763 cases [14]. The preparedness and response to the outbreak especially at the community and decision-making (state) levels need further description and consideration due to the absence of any previous assessment studies. This study aimed to assess the level of readiness and response toward the 2019 dengue fever outbreak in Kassala at the state and federal levels.

## Methods

### Study design and settings

This exploratory descriptive cross-sectional study was conducted in Kassala state, Sudan from January 2020 to June 2020. This state is characterized by the abundance of gardens and orchards and the diversity of nature. The state’s total area is 55,500 square kilometers and the estimated total population is 2,090,000 (according to the Sudan Central Bureau of Statistics Data Sheet 2018). Kassala State has 11 localities and the surveillance system covered 158 sentinel centers out of 364 of the health facilities in the state (43.4%). The Kassala locality is the center of Kassala State which consists of 6 sectors, and each sector has several neighbors.

### Study participants and sampling technique

The study population is the key informant personnel who work for surveillance, health promotion, vector control, outbreak response, case management, and curative medicine at Kassala State Ministry of Health. Overall, 7 key informant personnel were interviewed from the surveillance, health promotion, vector control, outbreak response, case management, and curative medicine departments of Kassala State MoH. For in-depth information, purposeful sampling was used for the key informant personnel in the departments that were directly linked to the dengue outbreak readiness and response in Kassala MoH (surveillance, health promotion, vector control, outbreak response, case management, and curative medicine departments).

### Inclusion and exclusion criteria

All key informative personnel of the targeted departments (surveillance, health promotion, vector control, outbreak response, case management, and curative medicine) who were in Kassala during the dengue outbreak and participated in the outbreak readiness and response processes were included in the study. Those who did or did not participate in the outbreak readiness and response processes were excluded from the study.

### Data collection

The interviewing data collection technique was used to collect information from the key personnel of the targeted departments at Kassala State Ministry of Health. In-depth interviews were conducted to fill out the open-ended questions and checklists developed by the WHO and related literature [8, 11]. The study instrument consisted of four parts, and each part had open-ended questions and checklists regarding outbreak management, surveillance, health Promotion, and vector control. Voice recorders were used with prior permission from the personnel to note and report the answers to the open-ended questions. The researcher conducted five interviews with the key respondents; each interview lasted an average of 40 min. Documentation of the interviews was performed by using a voice recorder and handwriting. These were implemented to assess the level of readiness and response and to reflect the capacity of institutions—public health authorities, health systems, and emergency response bodies.

### Data analysis

Interview contents were extensively analyzed about the main 4 dimensions affecting readiness and response (surveillance, vector control, outbreak management, and health promotion). Using thematic analysis, researchers have developed codes during the close examination of the narratives. Patterns of meaning and salient themes emerged from the texts. Words that strengthened or weakened each dimension were extracted from the key informants’ interviews.

## Results

### Key informant interview

#### Surveillance department

With concern to Kassala State readiness, the dengue outbreak was spotted by passive surveillance, and active surveillance has not yet been set up on the routine surveillance system. Dengue fever was laid on the list (A) of notifiable diseases according to the state and federal MOH classification (a single dengue case is considered an outbreak/no surveillance thresholds). During the outbreak, case definition publications (according to the WHO guidelines, available in both English and Arabic versions) were distributed to all health facilities of the state. Concerning laboratory readiness, there were no laboratory confirmations for all suspected dengue suspected cases, and no dengue rapid diagnostic tests were used in Kassala State during the outbreak. The FMoH supplied Kassala State with rapid diagnostic tests at the end of the outbreak. During the outbreak, all the blood samples (72 samples) were sent to the National Public Health Laboratory (Stack) in Khartoum, and they stopped receiving any more samples after 51 confirming samples out of 72 (as a percentage of 1.3% of the total cases). There was no risk assessment plan prepared before the outbreak emerged, and the state MoH only utilized the same scheme used in Kassala state for the chikungunya outbreak in 2018. Otherwise, no epidemiological, entomological, laboratory or geographical alerts were used. The work of epidemiological and entomological teams was in routine surveys, their reports were only combined during the outbreak periods.

The surveillance approaches that were used in the outbreak response were sentinel, community-based, and event-based surveillance. These three approaches were the main surveillance approaches normally used in Kassala State, and no syndromic surveillance or laboratory surveillance was used. Additionally, vector surveillance was not used or linked to routine surveillance. Regarding the private sector, private hospitals and polyclinics were included in the surveillance system, while private clinics and labs were not. In referring to the surveillance reports on the outbreak response, only 158 out of 362 (43.6%) health facilities reported on time to the state surveillance department as sentinel units. Until the end of the outbreak, there was no electronic system for reporting, and they conveyed the reports through active visits to health centers or by telephone calls. The analysis process of dengue case data was not conducted at the locality level, and the analysis process was conducted at the federal and state Ministry of Health.

Political issues were considered one of the major challenges for the surveillance system, which led to being late in the announcement of the outbreak and delayed the outbreak response from the state directorate and the FMoH, although the surveillance department at the state was notifying for one dengue case as an outbreak. Furthermore, the fragmentation of the health system and the various outbreak activities of the departments, in addition to the lack of testing equipment for confirming the cases or having any resources for intervention procedures led to poor outbreak response.

### Outbreak management and response (curative medicine & primary healthcare departments)

In connection with the outbreak readiness in Kassala, the health personnel were not sufficient at the beginning of the outbreak, the FMoH responded in November 2019 by sending more than 100 doctors to be trained for one month. During the outbreak, free drugs for dengue fever (analgesics and intravenous fluids) were available and prescribed freely for admitted patients in governmental healthcare facilities. Additionally, insecticide-treated nets (ITNs) were distributed throughout the whole of the state after the peak of the rainy season (after the outbreak commencement) and set to every bed throughout the state health facilities.

There was a contiguous plan for outbreak response after the minister of health was changed at Kassala State in the last quarter of 2019. This plan involved international nongovernmental organizations (INGOs), the central committee of Sudan doctors, and civil societies in counseling and orientation of the notification system. Additionally, the risk communication was transparent and applied with fewer restrictions.

Before the INGOs took part in the outbreak intervention (case management intervention and health promotion programs) in the middle of the outbreak period, all the response interventions were solely represented by the MoH. The civil societies and youth initiatives played a valuable role in the outbreak response, and they were formally involved as stakeholders in November 2019. However, governmental authorities such as those in the environmental and educational sectors did not collaborate or participate in the outbreak response with the health sector due to the lack of preestablished communication or cooperation.

The significant challenges faced by the curative medicine and primary healthcare departments were considered the lack of financial resources, trained personnel, and the absence of any routine procedures and activities before the outbreak occurrence. These factors affected the quality of response in the departments of the surveillance system and vector control and led to the occurrence of dengue outbreaks in 2019 less than one year after the chikungunya outbreak in 2018. On the other hand, it was suggested to set up a protocol for case management followed by orientation sessions for the healthcare workers and establishment of rural healthcare units for the outbreak because most of the cases with complications were from rural areas.

### Environmental health and vector control department

Concerning outbreak readiness, there were no specific resources for this outbreak, and most of the environmental health and vector control resources and equipment were obtained from the previous Chikungunya outbreak in 2018. Guidelines manual were available for working and supervision of the health promoters and vector control workers. The health promoter teams were trained on how to deliver educational messages to households and check their water containers, which could be considered nonchemical mosquito control measures. For chemical mosquito control, Abate 500 EC (with temephos as an active ingredient) was used as the main insecticide for vector control in this outbreak. This Abate 500 EC was checked before the outbreak for its effectiveness. On rare occasions, diesel is also used as a chemical method for vector control. The biological control was never used before in the state. Generally, there is no entomological laboratory to compare the types of virus present inside the vector and human beings in the state.

Regarding the environmental health and vector control response during the outbreak, there were 7 stations in the Kassala locality plus 4 stations in rural Kassala covered weekly. The entomological indices (Breteau, house, container, and pupae) were reported daily during the outbreak. All localities send their reports for vector control to the state level every week. The larval control consisted of two main parts: indoor and outdoor. In the indoor part, the health promoters made home visits to clean and dry the water containers and provided educational messages to households. The outdoor part included outdoor insecticides. Adult mosquito control was represented by a fogging spray using a fogging machine. This fogging spray was used according to the number of cases, so the area with a high number of cases reported was targeted to have a fogging spray. This spray was used for a total of eight weeks throughout the outbreak period. During the outbreak, no trained staff could disseminate spatial GIS maps to help in spraying and vector control. Moreover, the response activities involved a wide number of governmental facilities (such as schools) according to the available resources by inspecting and cleaning the water containers and then conducting a fogging spray while the schools were closed at the early stage of the outbreak.

The community was one of the challenges for environmental health and vector control, and some of them were unconvinced that the breeding sites of mosquitoes could be inside their homes. They heard and were aware of dengue, and some of them refused the health promoters to enter their homes. Additionally, the absence of entomological laboratories creates difficulties for the department in determining the types of viruses inside the vectors.

### Health promotion department

During the outbreak in Kassala State, there was no standard approach for community engagement. The major health promotion activities focus on “house-to-house visits” and mass media programs through television episodes including an interview with healthcare promoters. The health promotion plans focused on the behavior change, especially on water container dryness. In the middle of the outbreak, the health promotion team was changed. The new team conducted a small community survey related to health promotion activity. Then they changed the main message of the health promotion campaign from the old slogan “dry the sources of mosquito breeding sites twice a week” to the new slogan “If someone at home was infected by dengue, this is because you miss drying”, the new slogan had an indirect message with emotion. Furthermore, the youth initiatives played a major role as a third partner to overcome the community resistance and build community engagement by conducting mass campaigns and participating in blood donation, providing medication for case management, and sharing knowledge and awareness throughout the community. The mass campaigns of those youth innovations were designed to be as tents located in the local markets of Kassala locality, and each tent had a catchment area to be covered at the end of the program. These tents were equipped with posters, a sound system, and trained volunteers for implementing exhibitions, roleplays, lectures, and group discussions. Any educational messages used in these tents should be reviewed and approved by the state ministry of health first. In addition to that, all the health messages were translated into the local language of the different tribes of the Kassala locality. This program continued for 20 days.

In addition to the late response and poor community engagement, the obvious challenges faced by the health promotion department was community behavior. Some of the local citizens resisted the “house-to-house visit” approach and some of the households did not allow health promoters to check their underground water storage. A large number of state people showed a full reliance on the health promoters and saw them as “clerks” whose responsibility was to clean the water container. In addition, the lack of proper monitoring and evaluation processes for providing sustainable funds and resources for health promotion programs and projects can cause obstacles in the future.

### Outbreak readiness and response checklist

**Fig. 1:**
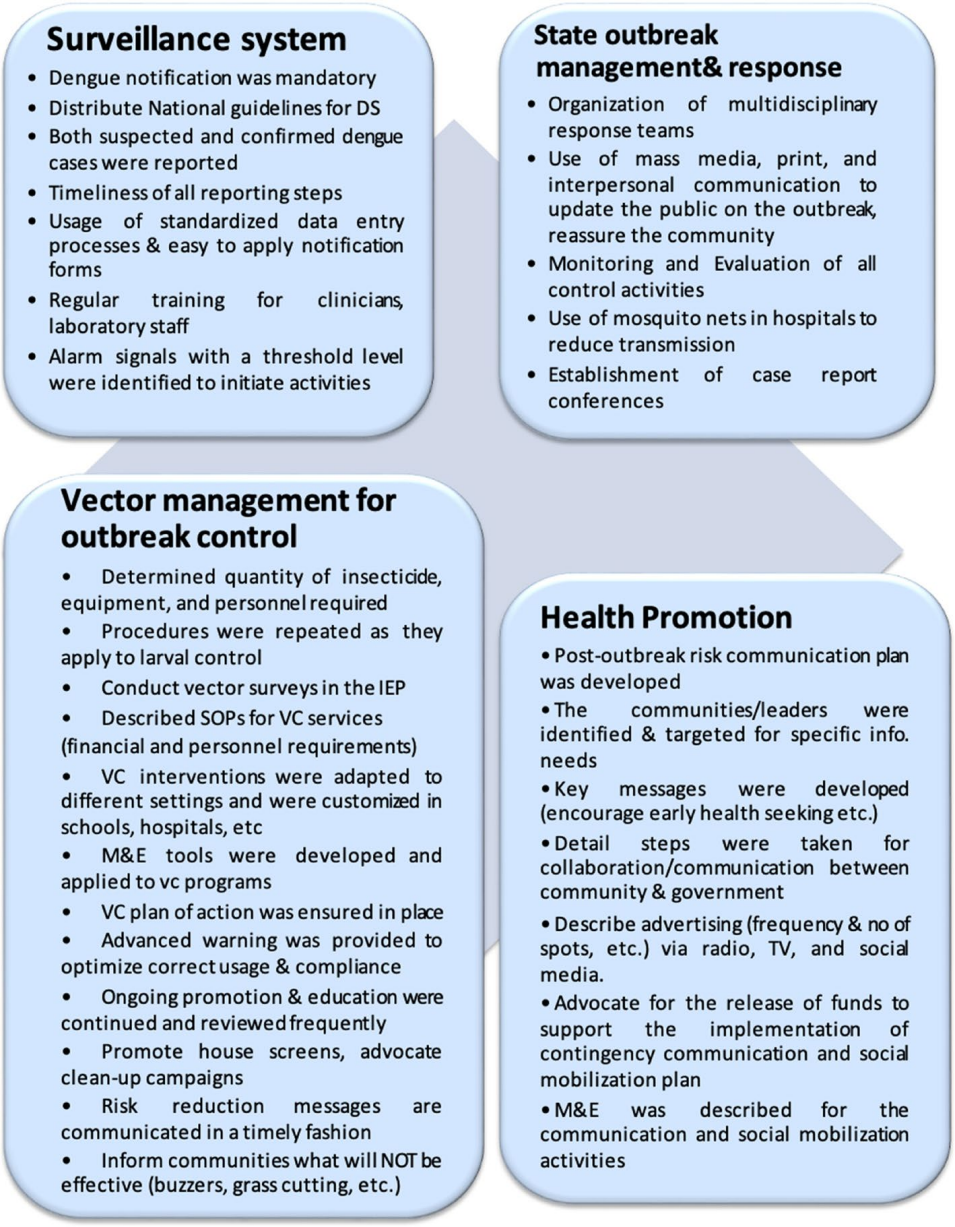
Summary of fulfillment elements of WHO checklist (DS: Dengue Surveillance, IEP: inter-epidemic period, M&E: Monitoring & Evaluation, VC: vector control)

**Fig. 2:**
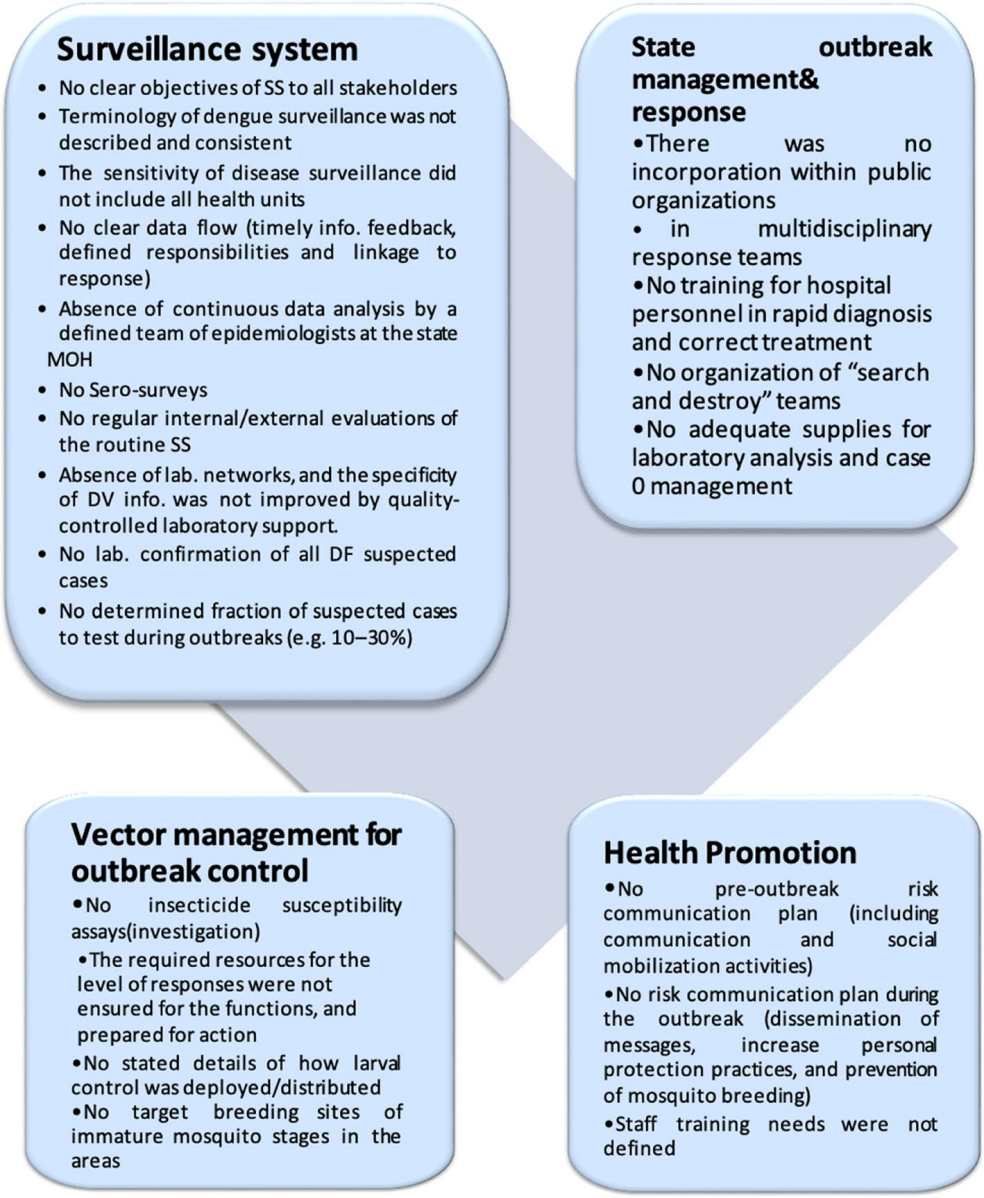
Summary of nonfulfillment elements of WHO checklist

Based on the respondents’ feedback on the study checklist elements related to outbreak management, surveillance, health promotion, and vector control. In this outbreak, the vector control and health promotion interventions were relatively matched with the WHO model contingency plan elements. Figure 1 demonstrates the summary of fulfillment items of the checklist. On the other hand, there was an obvious gap in the outbreak management activities and surveillance capabilities in this outbreak. Figure 2 reveals the nonfulfillment elements of the checklist.

## Discussion

Outbreak response has been recognized as a cycle containing many factors and elements such as community and partner engagement, capacity building, and providing operational connections to reach a level of structured and coordinated response. One objective of this study was to determine the readiness and responses at the state level during the dengue outbreak and provide a holistic and comprehensive concept of how surveillance, vector control, outbreak management, and health promotion plans and procedures were implemented at the time of the outbreak. The findings data gathered from the state-targeted departments and personnel were analyzed and compared to WHO documents and previous recommendations.

Surveillance is a critical component of any dengue prevention and control program because it provides the information necessary for risk assessment and program guidance, including epidemic response and program evaluation. The restricted routine surveillance at the state, with the absence of active surveillance, unprepared community-based surveillance, and limited sentinel surveillance introduces the fragility of the surveillance system with the underreporting of cases that undermining the ability of surveillance to include all health facilities and home treating cases. According to the WHO global strategy for dengue prevention, the surveillance system for dengue should be a part of the national health information system, with a set of core indicators monitored at various levels of the health administration, and data quality also needs to be monitored and assessed periodically [1]. These were not applied to the state surveillance system due to unharmonized effort and the absence of regular internal or external evaluations of the state routine surveillance system. There was a similarity with most findings of a study conducted by J. Harrington et al. [11] in using a passive surveillance system to detect the outbreak, lack of electronic systems, and absence of surveillance thresholds to define the outbreak. On the other hand, national guidelines for dengue surveillance which were distributed to all state health facilities may provide a good impact on strengthening case management and refreshing staff knowledge during the outbreak period. This may be considered a substitute for the hands-on training that showed favorable results during the outbreak in Brazil [15]. According to the surveillance department personnel’s interview, the political issue was considered one of the major barriers of action that may affect the effectiveness and the time of response from the state and federal authorities. This could be directly linked to the lack of accountability at all levels, which needs to be strict and functional to shorten the time delay between the onset of and response to an epidemic.

Generally, the dependence on weak fragmented passive surveillance systems provides meaningless reporting with an inaccurate number of cases, inadequate methods of notification, and weakness in the transfer of case information from the local to the central level for analysis, with a lack of local use of analysis data, which may lead to delay of response. In addition, laboratory surveillance needs to be strengthening for outbreak preparedness and response because the blood samples collected during the outbreak in Kassala were unrepresentative (1.3%) and did not demonstrate an accurate number of dengue cases or the other coinfections such as chikungunya fever and yellow fever.

Regarding outbreak management and according to the previous literature. The control and response activities for a dengue outbreak generally need to be multisectoral, multidisciplinary, and multilevel, requiring environmental, political, social, and medical inputs to be coordinated. In addition, outbreak planning must achieve governance over the response, especially by involving stakeholders and providing response monitoring details [11]. This has not occurred in this outbreak because it was solely represented by the Ministry of Health, without any active participation or engagement from other stakeholders. In this outbreak, there was an obvious neglect in stakeholder role documentation (“what” should be implemented and “who” shall be responsible) in outbreak response plans prior to or after the contiguous plan, which led to a failure to acknowledge the importance of intersectoral communication, a failure to recognize capacity, a lack of appropriate delegation, and a lack of accountability concerning activities. In addition, the outbreak management and response at Kassala State did not include any training of hospital personnel in rapid diagnosis and correct treatment. This may also negatively affect the state response due to its prerequisite in the interepidemic period according to the literature [9]. Furthermore, the number of health staff was not sufficient to cover all the cases during the outbreak, and the response from the Federal Ministry of Health was late (in the urgent stage) to supply them by health care workers. Therefore, investment in human resources must come before the outbreak. While the insecticide-treated nets (ITNs) were set to every bed throughout the health facilities in Kassala State during the outbreak, they showed an obvious reduction in cases and prevented the spread of dengue within a hospital according to a study in India [16].

Effective vector control measures are critical to achieving and sustaining the reduction of morbidity attributable to dengue. The environmental health and vector control at the state showed satisfactory efforts during the outbreak in comparison with available resources, by repeating the vector/larvae control procedures, adapting the interventions to different settings, and customizing the programs to involve schools, hospitals, etc. Furthermore, there were daily entomological indices reports (Breteau, house, container, and pupae), and this finding was similar to some reports of a study conducted by Harrington and co-authors [11] that monitored the entomological indices. Additionally, the outdoor space spraying of insecticides (fogging spray) was involved in the outbreak response similar to the majority of literature reports. Combined vector control interventions (larvicides and space spray) were used in this outbreak, which is previously recommended and used by most reports [9]. Overall, the state vector control activities need to engage the community to help in targeting the breeding sites of immature mosquito stages in the areas, in addition to using electronic software such as GIS in vector control procedures.

According to the WHO global strategy for dengue prevention, dengue cases and dengue deaths can be reduced only through the behavioral actions of those responsible for designing and implementing dengue prevention and control programs and by the adoption of risk reduction and health protection behaviors through the populations at risk [1]. The health promotion procedures had two stages during the outbreak. The first stage involved the classical implemented approach at the state during recent years, such as “house-to-house visits” and mass media (television) programs. However, these approaches did not give an obvious outcome during the outbreak, and they brought an apparent community resistance according to the new head of the health promotion department. Therefore, during the second stage (which took approximately 20 days), the Ministry of Health conducted mass campaigns and involved youth initiatives as a third partner to overcome community resistance. This second intervention at the state includes relatively more community engagement activities than the first, which provides an obvious outcome in dengue case reduction. This circumstance met the core findings reported by [9] which revealed that all studies that incorporated community organizations within their outbreak management organizational structure achieved successful outbreak control.

Last, the occurrence of this dengue outbreak in Kassala State after 4 months of the chikungunya outbreak with 19,902 cases [14] and stay for five months may indicate the weakness of preparedness and readiness of the health system in the state.

### Limitations

There were several limitations in this study. Generally, the lack of a previous study on the same subject made discussion of the results more difficult. In addition, the retrospective nature of the study, underreporting and poor health information system, and absence of a formula to adjust for background endemicity were considered common limitations in the study.

## Conclusions

Overall, mosquito-borne diseases, such as dengue fever—can be considered endemic diseases in Kassala State. For that reason, the federal and state MOH may consider boosting their readiness plans and providing both diagnostic and curative measures to enhance the level of response to dengue fever cases. Our study revealed that most of the cases of this outbreak were reported based on clinical features and the actual number of dengue fever cases during this outbreak may be three- or fourfold larger than the reported cases due to weak surveillance systems. Therefore, this outbreak was considered one of the largest outbreaks reported in 2019 in Sudan. The outbreak readiness could be regarded as below standard, mainly in the surveillance system and laboratory diagnostic facilities. Moreover, the heterogeneous outbreak response could be considered a weakening outbreak response due to the absence of intersectoral collaboration with a regulatory framework in terms of financial and operational participation, in addition to poor community participation in operational activities.

- It is recommended that the state MoH of Kassala and the FMoH of health may consider linking the various departments’ activities to outbreak readiness and response, in addition to improving intersectoral collaboration before and during the outbreak occurrence.
- The surveillance system should be upgraded by promoting community-based surveillance, expanding the sentinel centers, and establishing laboratory surveillance along with active surveillance to provide early warning information for timely rapid response.
- Routine vector control activities should be continued regularly, and the locations selected for vector surveillance should be reviewed periodically to meet the latest needs. Moreover, an entomological laboratory is needed to compare the types of viruses present inside vectors and humans.
- The preventive measures of health promotion and vector control should be expanded to provide a space for community participation and cooperation in all sustainable preventive measures for mosquito control.
- Behavioral changes should be encouraged in health promotion plans to break the transmission of the disease including appropriate and safe vector control measures.

### Supplementary Information

**Additional file 1.**

## Data Availability

All data generated or analyzed during this study are included in this published article.

## Abbreviations

DF: Dengue Fever
GIS: Geographic Information System
INGOs: International Non-governmental Organizations
MoH: Ministry of Health
WHO: World Health Organization
