# Supplementary material for: Assessment of the readiness and response toward the dengue fever outbreak (2019) in Sudan: a qualitative exploration
Source: BMC Public Health. 2023 Oct 30;23:2122. doi: 10.1186/s12889-023-17020-9 (PMC10614372; doi:10.1186/s12889-023-17020-9)
Supplement: Supplementary file 1 — Additional file 1. [file 12889_2023_17020_MOESM1_ESM.docx]

**Readiness and response checklist**

| **Elements and characteristics of an adequate surveillance system (WHO)** | |
| --- | --- |
| 1 | Objectives of the surveillance system(s) was clear to all stakeholders |
| 2 | Terminology of dengue surveillance was described and consistent |
| 3 | Dengue notification was mandatory |
| 4 | National guidelines for dengue/disease surveillance was distributed |
| 5 | Both suspected and confirmed dengue cases were reported |
| 6 | Timeliness of all reporting steps were optimized |
| 7 | The sensitivity of disease surveillance for early including the private sector, all health units including outpatient departments and all age groups. |
| 8 | Usage of standardized data entry processes & easy to apply notification forms |
| 9 | Clear data flow (timely information feedback, defined responsibilities and linkage to response that should be in place) |
| 10 | Continuous data analysis including the lowest possible level of the health system by a defined team of epidemiologists should be ensured |
| 11 | Sero-surveys (assess the level of underreporting etc) |
| 12 | Regular internal and external evaluations of the routine surveillance system |
| 13 | The specificity of dengue information was improved by quality-controlled laboratory support. (Laboratory networks are crucial) |
| 14 | Laboratory confirmation of all dengue suspected cases |
| 15 | Small fraction of suspected cases was tested during outbreaks (e.g. 10–30%) |
| 16 | Regular training for epidemiologists, clinicians, laboratory staff was ensured |
| 17 | Alarm signals with a threshold level was identified to initiate activities |
| **Outbreak management and response checklist (WHO)** | |
| 1 | Organization of multidisciplinary response teams |
| 2 | Incorporation of public organizations in multidisciplinary response teams |
| 3 | Use of mass media, print and interpersonal communication to update the public on the outbreak, reassure the community that actions are being taken |
| 4 | Monitoring and Evaluation of all control activities |
| 5 | Organization of “search and destroy” teams |
| 6 | Training of hospital personnel in rapid diagnosis and correct treatment |
| 7 | Provide adequate supplies for laboratory analysis and case 0 management |
| 8 | Use of mosquito nets in hospitals to reduce transmission |
| 9 | Establishment of case report conferences |
| **Vector management for outbreak control checklist (WHO)** | |
| 1 | The quantity (number of houses treated, coverage, treatment frequency) of insecticide, equipment and personnel required were determined |
| 2 | These procedures were repeated as they apply to larval control |
| 3 | Insecticide susceptibility assays(investigation) was frequently conducted to ensure that insecticides being used remain effective |
| 4 | Conduct vector surveys in the inter-epidemic period |
| 5 | The required resources for the level of responses were ensured for the functions, and prepared for action |
| 6 | The details of how larval control was deployed / distributed was stated |
| 7 | The SOPs for vector-control services, including minimum financial and personnel requirements were described |
| 8 | The different vector-control interventions adapted to different settings or productive container types were described |
| 9 | The vector control programmes were customized in schools, hospitals, etc |
| 10 | M&E tools was developed and applied for vector control programmes |
| 11 | Ensure that a vector control plan of action is in place |
| 12 | Advanced warning was provided to optimize correct usage & compliance, if any planned vector control tools/strategies haven’t been used in the community |
| 13 | Ongoing promotion and education were continued and reviewed frequently |
| 14 | Target breeding sites of immature mosquito stages on the area particularly and deliver an impact on the adult vector population, approx. 2–3 weeks later |
| 15 | Promote house screens, advocate clean-up campaigns |
| 16 | Ensure risk reduction messages are communicated in a timely fashion and that the messages reflect the status of the outbreak |
| 17 | Inform communities what will NOT be effective (buzzers, grass cutting, etc.) |
| **Health promotion and social mobilization checklist (WHO)** | |
| 1 | Pre-outbreak risk communication plan was developed (including communication and social mobilization activities) |
| 2 | Outbreak risk communication plan was developed (dissemination of messages, increase personal protection practices and prevention of mosquito breeding) |
| 3 | Post-outbreak (recovery phases) risk communication plan was developed (dissemination of messages informing affected populations the outbreak is over, acknowledging their efforts to contain the outbreak) |
| 4 | The communities/leaders were identified & targeted for specific info. needs |
| 5 | Staff training needs were defined |
| 6 | Key messages were developed (encourage early health seeking and immediate care for patient who have any one of the dengue warning signs…) |
| 7 | Detail steps were taken for collaboration/communication between community and government (e.g. community meetings, stakeholder meetings etc.) |
| 8 | Describe advertising (number of spots, frequency of each spot, length of time each spot will air) via radio, television, newspapers, social media, etc. |
| 9 | Advocate for release of funds to support implementation of contingency communication and social mobilization plan |
| 10 | M&E was described for the communication and social mobilization activities |
